# Acetabular labral tear is associated with high pelvic incidence with or without femoroacetabular impingement morphology

**DOI:** 10.1007/s00167-022-06881-z

**Published:** 2022-01-31

**Authors:** Hyuck Min Kwon, Byung-Woo Cho, Sungjun Kim, Ick-Hwan Yang, Kwan Kyu Park, Nak-Hoon Son, Woo-Suk Lee

**Affiliations:** 1 Department of Orthopedic Surgery, Yongin Severance Hospital, Yonsei University College of Medicine, Seoul, Korea; 2 Department of Orthopedic Surgery, Severance Hospital, Yonsei University College of Medicine, Seoul, Korea; 3 Department of Radiology, Gangnam Severance Hospital, Yonsei University College of Medicine, Seoul, Korea; 4 Clinical Research (Biostatistician), Yongin Severance Hospital, Yonsei University College of Medicine, Seoul, Korea; 5 Department of Orthopedic Surgery, Gangnam Severance Hospital, Yonsei University College of Medicine, 211 Eonju-ro, Gangnam-gu, Seoul, 06273 Korea

**Keywords:** Acetabular labral tear, Pelvic incidence, Femoroacetabular impingement

## Abstract

**Purpose:**

The aim of this study was to investigate the association between pelvic sagittal parameters and acetabular labral tears.

**Methods:**

Three-hundred and sixty-five patients (449 hips) who underwent magnetic resonance imaging (MRI) or magnetic resonance arthrogram (MRA) for hip pain were enrolled in this study. Pelvic sagittal parameters, including the pelvic incidence, pelvic tilt, and sacral slope, were measured with a standing lumbosacral lateral radiograph. All subjects were divided into two groups according to the presence or absence of radiologic acetabular labral tears and compared. Furthermore, the two groups were divided into subgroups according to whether femoroacetabular impingement (FAI) morphology was present or not and compared.

**Results:**

Pelvic incidence was greater in the labral tear group than in the non-labral tear group (52.3° ± 8.2° versus 47.1° ± 6.8°, *p* < 0.001). After accounting for potentially confounding variables, we found that higher age (odds ratio 1.04, 95% confidence interval [CI] 1.02 to 1.06, *p* = 0.001), FAI (odds ratio 15.11, 95% CI 7.43 to 30.75, *p* < 0.001), and high pelvic incidence (odds ratio 1.13, 95% CI 1.09 to 1.17, *p* < 0.001) were independently associated with acetabular labral tear. When only the patients without FAI (308 hips) were divided into groups with and without acetabular labral tear, we found that higher age (odds ratio 1.03, 95% CI 1.01 to 1.06, *p* = 0.008) and high pelvic incidence (odds ratio 1.15, 95% CI 1.11 to 1.19, *p* < 0.001) were independently associated with acetabular labral tear.

**Conclusion:**

Acetabular labral tear is associated with high pelvic incidence with or without FAI morphology.

**Level of evidence:**

III.

## Introduction

Even though differential diagnosis is difficult due to complex pathological structure and acetabular dysplasia and ligamentous laxity might be the cause of groin or hip pain, acetabular labral tears have been reported as the cause of 22–55% of cases of groin or hip pain due to the location of pain-sensing free nerve endings in the labrum [26, 34, 36]. Although several studies have reported a high prevalence of asymptomatic acetabular labral tears in magnetic resonance imaging (MRI) [35, 40], some etiologies of acetabular labral tear have been investigated. Femoroacetabular impingement (FAI) is a bony morphology (cam or pincer type) and abnormal articulation of the femoral head and acetabulum that is associated with acetabular labral tears [28, 29]. FAI is considered one of the primary predisposing factors to acetabular labral tear due to impinging the anterior–superior portion of the labrum [17]. However, symptomatic acetabular labral tears are known to be common in patients without FAI as well as those with FAI [3, 25]. It is also well known for degenerative acetabular labral tears to be associated with degenerative changes of the hip joint [2, 36]. Some acetabular labral tears could be related to acetabular trauma and traumatic labral tears among athletes [5].

The spinopelvic sagittal parameter of pelvic incidence (PI) is found to be in the upright position by determining the shape and orientation of the lower limbs and spine, which is independent of the patient position and pelvic orientation [18]. It is defined as the angle between the line perpendicular to the midpoint of the sacral plateau and the line from this point to the center of the femoral head [22], and it is commonly measured on standing lateral lumbosacral radiograph images. Due to functional hip anatomy and the interrelationship among lumbar spine, pelvis, and hip kinematics, the impact of the PI angle with mechanical stresses on the hip joint is well recognized clinically [14, 20]. Some studies have determined that higher PI among younger individuals may contribute to the development of hip osteoarthritis due to biomechanical adaptations of the pelvis and decreased femoral head coverage [11, 18]. Some studies have also determined that patients with the cam-type or pincer-type FAI have smaller PI values than the healthy population [10]. Although this pelvic sagittal parameter influences the load across the hip joint and acetabular labrum, the effect of PI on acetabular labral tears is not well understood, especially in patients without FAI.

The purpose of this study was to investigate the association between pelvic sagittal parameters and acetabular labral tears. Based on the findings of previous studies mentioned above that abnormal pelvic sagittal parameters increase the mechanical forces applied to the hip joint, we hypothesized that abnormal pelvic sagittal parameters would be associated with acetabular labral tears. The hypotheses of the current study were that assessment of the association between pelvic sagittal parameters and acetabular labral tears in patients with hip pain or groin pain would be helpful in evaluating the acetabular labral tears.

## Materials and methods

**Fig. 1:**
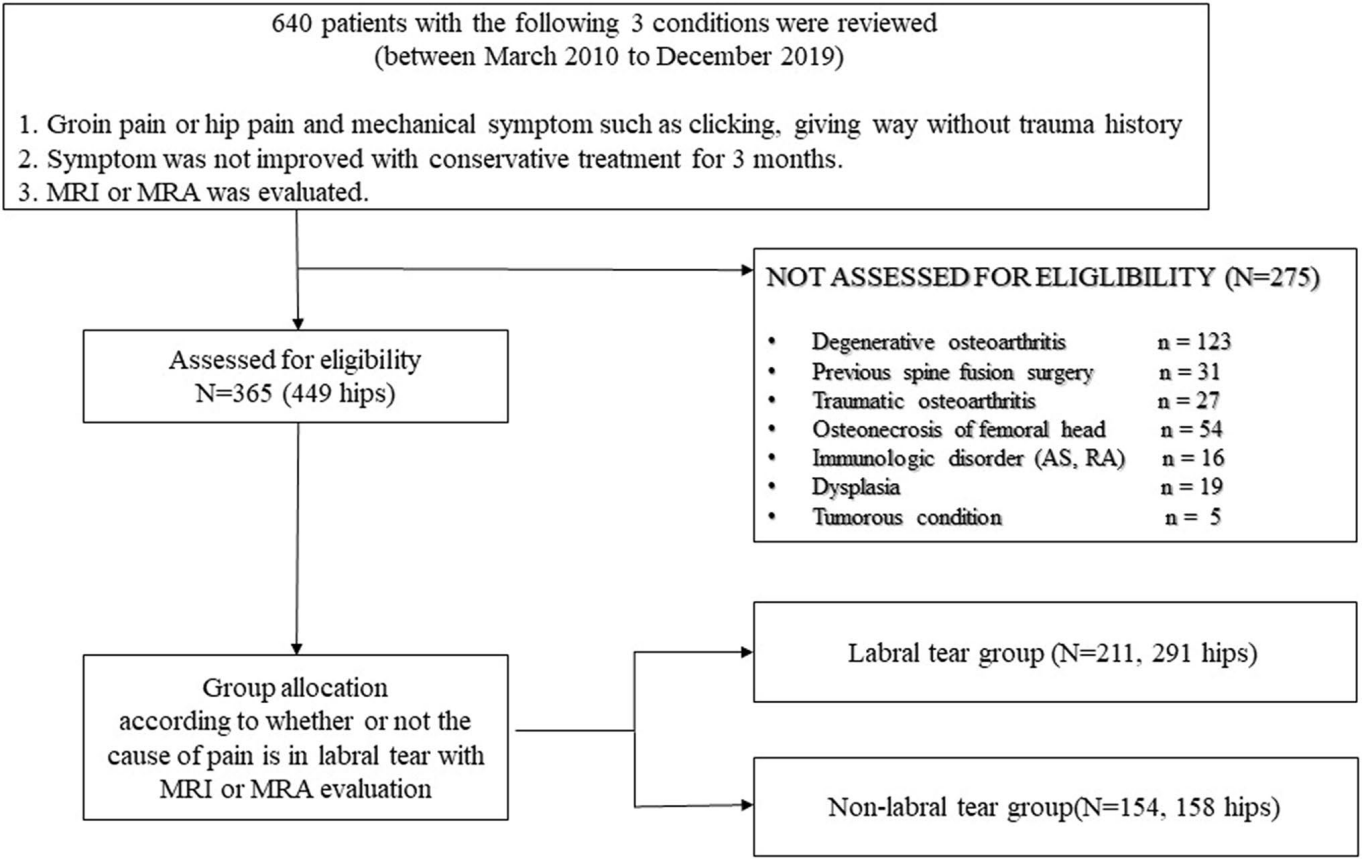
Flowchart of patient inclusion in the study

From March 2010 to December 2019, the Institutional Review Board (IRB)-approved database of 640 patients (Institutional Review Board of Yongin Severance Hospital, IRB # 9-2020-0051) who met the following three criteria in an outpatient clinic visit was retrospectively reviewed: (1) groin pain or hip pain and a mechanical symptom, such as clicking or giving way without trauma history; (2) symptom not improving with 3 months of conservative treatment; and (3) plain radiography (anteroposterior pelvis or hip radiograph, frog-leg lateral radiograph, and standing lumbosacral lateral radiograph) and MRI or magnetic resonance arthrogram (MRA). Only patients between the ages of 18 and 60 years were included. Patients were excluded if they had any of the following: a history of previous ipsilateral hip surgery or spine fusion surgery, or radiographic abnormalities, such as degenerative osteoarthritis (Tönnis Grade ≥ 1, which corresponds to increased sclerosis of the head and acetabulum, slight joint space narrowing, and slight lipping at the joint margins) [7]; traumatic osteoarthritis; osteonecrosis of the femoral head; rheumatologic disorder, such as ankylosing spondylitis or rheumatic arthritis; or a tumorous condition. After applying the above-mentioned criteria, 365 patients (449 hips) were finally included in this study (Fig. 1). Of the 449 images in this study, only nine images had MRA images that were obtained after 10–15 mL of a 2 mmol/L diluted solution of gadopentate-dimeglumine was injected intraarticularly, and most were 3.0 T MRI.

### Radiologic assessment

Radiologic acetabular labral tear of patients was confirmed by MRI or MRA. An acetabular labral tear on the MR image was defined as an irregular labrum shape, a non-triangular labrum, a thickened labrum with no labral recess, a labrum with increased signal intensities on the T1 images, or labral detachment from the acetabular labrum [30]. All subjects were divided into two groups according to the presence or absence of radiologic acetabular labral tears. Furthermore, MRI or MRA and plain radiography were used to measure the alpha angle, lateral center–edge angle, typical pistol grip deformity, crossover sign, posterior wall sign, and ischial spine sign. The alpha angle was defined as the angle between the bisection of the femoral axis with a line connecting the center of the femoral head to the point where asphericity began in the oblique axial view on MRI or frog-leg lateral radiography [31, 39]. A pistol grip deformity, crossover sign, posterior wall sign, and ischial spine sign on a standard anterior–posterior hip plain radiography were evaluated [44]. The lateral center–edge angle was measured by drawing 3 lines on the anterior–posterior hip plain radiography: (1) a horizontal line connecting the bilateral inferior of teardrops, (2) a line through the center of the femoral head, perpendicular to line 1, and (3) a line through the center of the femoral head, passing through the most superolateral point of the acetabular sourcil. The angle created by lines 2 and 3 is the lateral center–edge angle [44]. The anterior center–edge angle was measured by drawing 2 lines on the false-profile view: (1) a vertical line through the center of the femoral head and (2) a line connecting the center of the femoral head and the most anterior point of the acetabular sourcil. The angle created by these two lines is the anterior center–edge angle [7]. On the basis of the above-mentioned radiologic findings, these three types of FAI morphology were considered: (1) cam type, characterized by the presence of the typical pistol grip deformity or alpha angle > 55°, (2) pincer type, characterized by the presence of a crossover sign or an lateral center–edge angle > 40°, and (3) mixed type, meeting the radiographic criteria for both cam and pincer types [1, 31, 42]. The acetabular labral tear and non-acetabular labral tear groups were each divided into subgroups according to whether or not FAI morphology was present.

The synovial herniation pit that is formed by herniation of soft tissue through erosion or perforation at the reactive interface area in the femoral neck was investigated using MR imaging [33]. A paralabral cyst in both intra- and extra-articular locations was defined as a well-defined fluid signal abnormality in direct contact with the acetabular labrum on MR imaging [21]. The presence of acetabular chondral pathology such as cartilage denudation or full-thickness cartilage defects was investigated during the MR imaging evaluation. Cartilage denudation was defined by MR findings of linear high signal intensity paralleling the subchondral bone plate within to the acetabular cartilage with chondral surface irregularity, and full-thickness cartilage defects were defined by extending to the subchondral bone [23, 32].

**Fig. 2:**
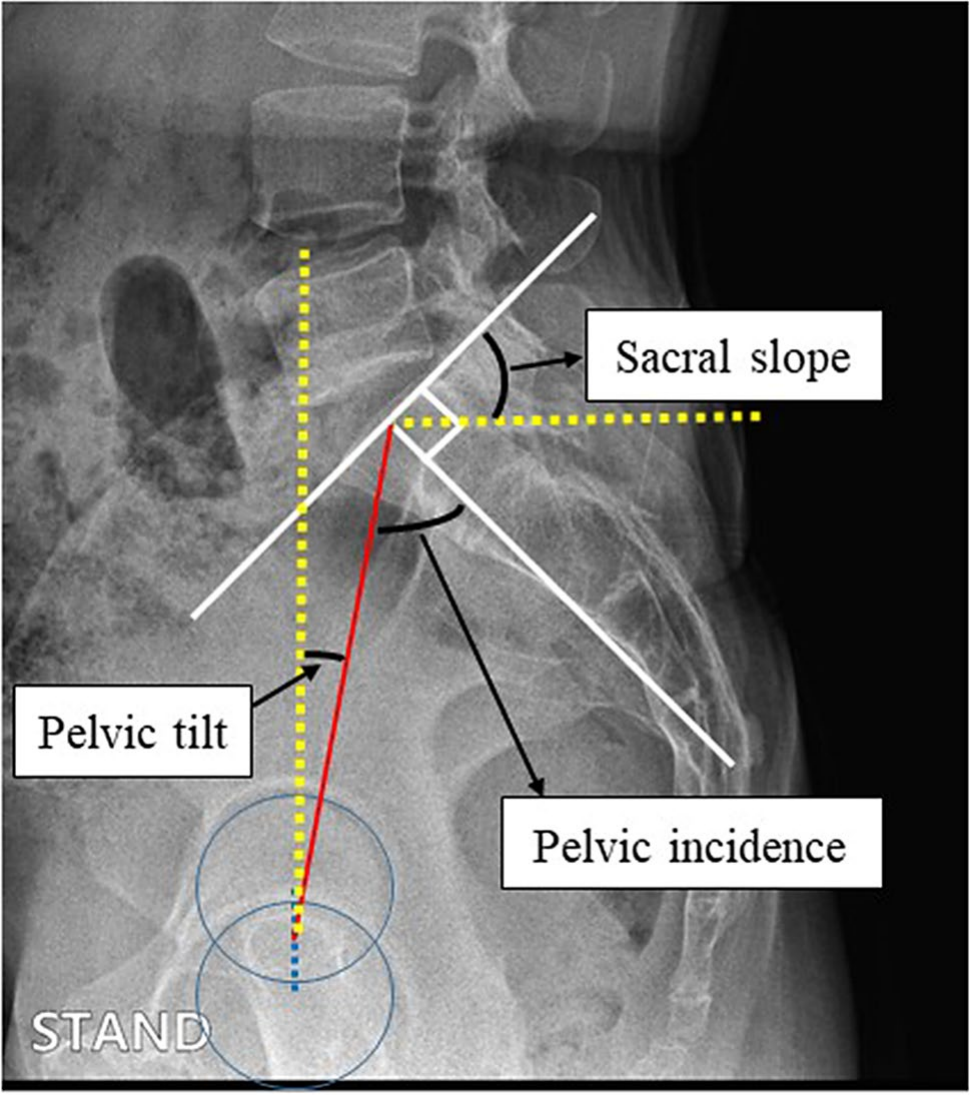
Pelvic sagittal parameters based on standing plain radiography

The pelvic sagittal parameters, including pelvic incidence, pelvic tilt, and sacral slope, were measured for all patients with a standing lumbosacral lateral radiograph (Fig. 2). Pelvic incidence was measured as the angle between the line perpendicular to the midpoint of the sacral plateau and the line from this point to the center of the femoral head [18]. Pelvic tilt was measured as the angle between the line joining the midpoint of the coxofemoral joint’s axis to the center of the S1 endplate and the vertical reference line. Sacral slope was measured as the angle between the line tangential to the superior endplate of S1 and the horizontal plane. All measurements were performed by one radiologist and one orthopedic surgeon. All of the measured angles were allowed one decimal digit. The degree of measurement reliability was evaluated using intraclass correlation coefficients (ICC). Calculation of the ICC was performed by one experienced radiologist and one experienced orthopedic surgeon. For the ICC, values less than 0.2 were considered to indicate poor agreement; 0.21–0.40, fair agreement; 0.41–0.60, moderate agreement; 0.61–0.80, good agreement; and above 0.80, excellent agreement [19]. The ICC for both intraobserver reliability and interobserver reliability were greater than 0.85. In particular, the presence of acetabular labral tear, the presence of FAI morphology, and the ICC of pelvic incidence were 0.915, 0.936, and 0.867, respectively.

### Statistical analysis

Chi-square test and *t* test were performed to compare the labral tear and non-labral tear groups. A multivariate logistic regression test with 95% CIs was performed to analyze the independent risk factors for acetabular labral tear. Statistical analyses were performed using the SPSS software for Windows (Version 20.0, SPSS, Chicago, IL, USA), and *p* values of less than 0.05 were considered significant. The statistical software G*Power (version 3.1.9.7; Heinrich Heine Universität Düsseldorf, DE) was used for sample size calculation. A total of 153 subjects were required to perform logistic regression analysis using a power of 0.95 and an alpha error of 0.05, according to a pilot study that included 50 patients; the sample size of the present study satisfied this requirement.

## Results

Of 449 symptomatic hips that did not respond to conservative treatment, acetabular labral tears in 291 hips (64.8%) were confirmed by MRI or MRA. Among them, when only 308 hips without FAI were analyzed, acetabular labral tears were confirmed in 164 hips (53.2%). When the 141 hips with FAI were analyzed, acetabular labral tears were confirmed in 127 hips (90%). As in previous studies [4, 8], FAI was confirmed to be a predisposing factor for acetabular labral tears due to its impinging on the anterior–superior portion of the labrum in bony deformity and abnormal articulation of FAI [17]. Additionally, the proportion of bilateral symptoms in the labral tear group was high (labral tear group vs. non-labral tear group in all patients: 38% vs. 3%; labral tear group vs. non-labral tear group in patients with without FAI: 34% vs. 1%).

Pelvic incidence was greater in the labral tear group than in the non-labral tear group (52.3° ± 8.2° versus 47.1° ± 6.8°, *p* < 0.001). Additionally, there were significant differences in age, sacral slope, pelvic tilt, FAI (cam or pincer morphology), herniation synovial pit, and cartilage denudation between the labral tear group and the non-labral tear group (Table 1). After accounting for potentially confounding variables like sex, BMI, FAI, cartilage denudation, and synovial herniation pit, we found that higher age, FAI, and high pelvic incidence were independently associated with acetabular labral tear (Table 2).

**Table 1:** Comparison of clinical patient data between the labral tear group and non-labral tear group

Characteristic	Labral tear group (*N* = 211, 291 hips)	Non-labral tear group (*N* = 154, 158 hips)	*p* Value
Male patients (%)	139 hips (48%)	71 hips (45%)	n.s
Age (years)	53.3 ± 11.7	48.4 ± 11.6	0.000
BMI (kg/m^2^)	23.4 ± 2.7	23.3 ± 2.4	n.s
Bilateral hip pain	80 patients (38%)	4 patients (3%)	0.000
Symptom duration (months)	8.7 ± 3.8	8.1 ± 3.6	n.s
Location of labral tears			
Anterior–superior	247/291 (84.9%)		
Posterior–superior	44/291 (15.1%)		
Pelvic sagittal parameter			
Pelvic incidence (°)	52.3 ± 8.2	47.1 ± 6.8	0.000
Sacral slope (°)	37.3 ± 7.7	35.6 ± 5.8	0.014
Pelvic tilt (°)	15.1 ± 6.6	11.5 ± 4.8	0.000
Radiologic measurement			
Lateral CEA (°)	28.1 ± 5.7	27.9 ± 3.3	n.s
Anterior CEA (°)	38.2 ± 7	38.1 ± 4.5	n.s
Alpha angle (°)	53.5 ± 6.3	48.2 ± 4.2	n.s
Femoroacetabular impingement	127 hips (44%)	14 hips (9%)	0.000
Cam type, *N* (%)	92 hips (32%)	12 hips (8%)	0.000
Pincer type, *N* (%)	22 hips (8%)	2 hips (1%)	0.000
Mixed type, *N* (%)	13 hips (5%)	0 hips (0%)	
Normal, *N* (%)	164 hips (56%)	144 hips (91%)	
Herniation synovial pit, *N* (%)	35 hips (12%)	7 hips (4%)	0.009
Cartilage denudation, *N* (%)	46 hips (16%)	6 hips (4%)	0.000

Data are presented as the mean ± standard deviation

*BMI* body mass index, *CEA* center–edge angle

**Table 2:** Results of multivariate logistic regression analyses of independent risk factors of acetabular labral tear in all patients

Factor	Multivariate analysis	
	OR (95% CI)	*p* Value
Age	1.039 (1.016–1.062)	0.001
Sex	1.492 (0.886–2.514)	n.s
BMI	0.980 (0.892–1.077)	n.s
FAI (cam, pincer, or mixed deformity)	15.227 (7.494–30.941)	0.000
Pelvic incidence	1.128 (1.091–1.168)	0.000
Cartilage denudation	2.074 (0.774–5.556)	n.s
Synovial herniation pit	2.673 (0.915–7.806)	n.s

*BMI* body mass index, *FAI* femoroacetabular impingement, *OR* odds ratio, *CI* confidence interval

When only the patients without FAI (308 hips) were divided into groups with and without acetabular labral tear, pelvic incidence was greater in the labral tear group than in the non-labral tear group (54.8° ± 7.6° versus 46.8° ± 6.9°, *p* < 0.001). Additionally, there were significant differences in age, sacral slope, pelvic tilt, lateral center–edge angle, anterior center–edge angle, herniation synovial pit, and cartilage denudation between the labral tear group and the non-labral tear group (Table 3). After accounting for the potentially confounding variables, we found that higher age and high pelvic incidence were independently associated with acetabular labral tear. No differences in symptom duration, sex, or BMI were found between the two groups (Table 4).

**Table 3:** Subgroup comparison of clinical data between the labral tear group and non-labral tear group in patients without femoroacetabular impingement

Characteristic	Labral tear group (*N* = 122, 164 hips)	Non-labral tear group (*N* = 142, 144 hips)	*p* Value
Male patients (%)	53 hips (32%)	62 hips (43%)	n.s
Age (years)	54.4 ± 11.2	48.7 ± 11.6	0.000
BMI (kg/m^2^)	23.3 ± 2.8	23.3 ± 2.4	n.s
Bilateral hip pain	42 patients (34%)	2 patients (1%)	0.000
Symptom duration (months)	8.8 ± 3.9	8.1 ± 3.4	n.s
Location of labral tears			
Anterior–superior	143/164 (87.2%)		
Posterior–superior	21/164 (12.8%)		
Pelvic sagittal parameter			
Pelvic incidence (°)	54.8 ± 7.6	46.8 ± 6.9	0.000
Sacral slope (°)	38.6 ± 8.1	35.7 ± 5.8	0.000
Pelvic tilt (°)	16.5 ± 7.2	11.3 ± 4.7	0.000
Radiologic measurement			
Lateral CEA (°)	26.8 ± 5.5	27.9 ± 3.2	0.040
Anterior CEA (°)	35.6 ± 5.9	38.0 ± 4.3	0.011
Alpha angle (°)	47.8 ± 5.5	47.4 ± 2.6	n.s
Herniation synovial pit, *N* (%)	12 hips (7%)	7 hips (5%)	0.009
Cartilage denudation, *N* (%)	21 hips (13%)	3 hips (2%)	0.000

Data are presented as the mean ± standard deviation

*BMI* body mass index, *CEA* center–edge angle

**Table 4:** Results of multivariate logistic regression analyses of independent risk factors of acetabular labral tear in patients without femoroacetabular impingement

Factor	Multivariate analysis	
	OR (95% CI)	*p* Value
Age	1.034 (1.009–1.059)	0.008
Sex	1.456 (0.816–2.597)	n.s
BMI	0.958 (0.864–1.061)	n.s
Pelvic incidence	1.145 (1.102–1.190)	0.000
Cartilage denudation	4.003 (1.002–15.996)	n.s
Synovial herniation pit	1.669 (0.484–5.965)	n.s

*BMI* body mass index, *OR* odds ratio, *CI* confidence interval

When all of the participants were divided into two groups (pelvic incidence > 55°, pelvic incidence < 55°) based on earlier studies [18, 38, 41], pelvic incidence greater than 55° had a sensitivity of 45.4%, specificity of 86.7%, positive predictive value of 86.3%, and negative predictive value of 71.3% for predicting acetabular labral tears in all of the patients (Table 5). In patients without FAI, pelvic incidence greater than 55° had a sensitivity of 59.8%, specificity of 87.5%, positive predictive value of 84.5%, and negative predictive value of 65.6% for predicting acetabular labral tears (Table 6).

**Table 5:** Analysis of pelvic incidence and acetabular labral tears in all patients (449 hips)

Pelvic incidence	Labral tear group	Non-labral tear group	Total
> 55°	132 hips	21 hips	153 hips
< 55°	159 hips	137 hips	296 hips
Total	291 hips	158 hips	449 hips
Statistics		Estimate	
Sensitivity		45.4%	
Specificity		86.7%	
Positive predictive value		86.3%	
Negative predictive value		71.3%

**Table 6:** Analysis of pelvic incidence and acetabular labral tears in patients without femoroacetabular impingement (308 hips)

Pelvic incidence	Labral tear group	Non-labral tear group	Total
> 55°	98 hips	18 hips	116 hips
< 55°	66 hips	126 hips	192 hips
Total	164 hips	144 hips	308 hips
Statistics		Estimate	
Sensitivity		59.8%	
Specificity		87.5%	
Positive predictive value		84.5%	
Negative predictive value		65.6%	

## Discussion

The principal finding of this study was that higher age and high pelvic incidence were associated with acetabular labral tears. In particular, subgroup analysis according to the presence or absence of FAI showed that higher age and high pelvic incidence were associated with acetabular labral tears in patients without FAI. After controlling other variables, higher age and high pelvic incidence were highly associated with acetabular labral tears. Additionally, pelvic incidence greater than 55° in a standing lumbosacral lateral radiograph predicts an increase in the possibility of acetabular labral tear in patients with hip pain who do not respond to conservative treatment. Because 48–95% of acetabular labral tears are associated with substantial damage to the acetabular cartilage, higher age and degeneration based on radiographic arthritic change are considered to be risk factors for acetabular labral tears [2, 9, 12, 27]. However, little is known about the effect of pelvic sagittal parameters such as PI on acetabular labral tears. High pelvic incidence was associated with acetabular labral tears after controlling for age, sex, BMI, FAI, cartilage denudation, and synovial herniation pit. Since high pelvic incidence and biomechanical adaptations may contribute to the excessive stress experienced by the hip joint and acetabular labrum, the evaluation of pelvic incidence in patients with hip pain could help to assess the possibility of acetabular labral tears [15, 16].

Although there is some controversy, pelvic incidence has been considered as a specific morphological parameter for each patient, since it does not change in the external pelvic position [24]. High pelvic incidence means theoretically increased lumbar lordosis, and in attempts to compensate from the increased mechanical loading of lumbar lordosis and maintain sagittal balance, high pelvic incidence is accompanied by posterior pelvic tilt [43]. This posterior tilt of the pelvis resulted in decreased femoral head anterior coverage by creating a more vertical articular surface of the acetabulum. In this way, excessive mechanical stress will be applied to the mainly anterior labrum of the acetabulum, which would increase the occurrence of acetabular labral tears [6, 13, 37]. In particular, 84.9% of the acetabular labral tears in this study were located in the anterior–superior portion of the labrum, which may be thought to be influenced by the increase in mechanical load to the anterior portion of the labrum and due to the high pelvic incidence. Therefore, we suggested in this study that high pelvic incidence could potentially be associated with the development of acetabular labral tears, especially in patients without FAI, even though FAI was confirmed to be the most predisposing factor of acetabular labral tears.

Previous studies have examined the influence of abnormal pelvic incidence (high or low pelvic incidence) on hip joints, resulting in conditions such as hip osteoarthritis, FAI, or osteonecrosis of the femoral head [10, 11, 18]. In particular, acetabular over-coverage is associated with FAI, and it is known to be related to acetabular labral tears [17]. Low pelvic incidence does not allow for modification of the pelvic motion, so amplified force is applied to the femoroacetabular joint, thus resulting in impingement and acetabular labral tears [10]. However, our investigations in the present study revealed that not only low pelvic incidence but also high pelvic incidence is associated with acetabular labral tears in patients with a symptomatic hip. Because abnormal spinopelvic parameters could affect hip joints, analysis of spinopelvic parameters and sagittal imbalance should not be underestimated or poorly determined by a hip surgeon.

There were several limitations to this study. First, this study had a retrospective design and a relatively small number of patients, which could be associated with a risk of bias in analysis. Moreover, due to the nature of the retrospective study, it was difficult to accurately compare with a true control group. In addition, higher age was a very important factor regardless of the presence of osteoarthritis in the acetabular labral tear, and this study was limited in that the two groups were not properly matched by age. Second, the subjects were only those who did not respond to 3 months of conservative treatment; however, the duration of symptoms varied from 3 to 18 months. Although there was no difference in symptom duration between the groups with and without acetabular labral tear, the various symptom durations may have influenced the outcome. In addition, it is possible that hip pain or groin pain does not indicate a labral tear but rather a symptom caused by internal snapping or extra-articular structure, and there was no confirmation process, such as injection, to determine whether pain of intra-articular origin was recognized. Third, since the present study was conducted on Koreans, it may be difficult to generalize to other populations. Additionally, acetabular labral tear in this study was based on the diagnosis on MRI or MRA, not arthroscopic confirmation. Therefore, there may be bias depending on whether MRI or MRA was used, and chondrolabral separation or intrasubstance tearing can lead to the overdiagnosis as acetabular labral tears.

## Conclusion

Acetabular labral tear is associated with high pelvic incidence with or without FAI morphology. The assessment of pelvis incidence and FAI morphology in patients with hip pain may help surgeons to evaluate acetabular labral tears.
